# Response to Schmidt et al.: Lower activity of cholesteryl ester transfer protein (CETP) and the risk of dementia: a Mendelian randomization analysis

**DOI:** 10.1186/s13195-024-01631-4

**Published:** 2024-12-19

**Authors:** Emma L Anderson

**Affiliations:** Division of Psychiatry, University College London, 149 Tottenham Court Road, W1T 7NF London, UK

**Keywords:** Cholesteryl Ester transfer protein, CETP, Dementia, Alzheimer’s disease, Vascular dementia, Mendelian randomization, Drug target

## Abstract

A recent paper concluded that cholesteryl ester transfer protein (CETP) inhibition may be a viable target to treat dementia, based on human genetic evidence of a protective effect of target inhibition on risk of Lewy body and Parkinson’s dementia. Alzheimer’s disease, which is by far the most prevalent cause of dementia (around 80% of all dementia cases) was not included as an outcome. Evidence shows CETP inhibition is unlikely to affect Alzheimer’s risk and may even potentially modestly increase risk. There is also little evidence to support an effect of CETP inhibition on all-cause or vascular dementia. Thus, CETP inhibition is unlikely to be a viable target to treat the most prevalent causes of dementia.

**Supplementary Information:**

The online version contains supplementary material available at 10.1186/s13195-024-01631-4.

## Response

I read with interest the recent study by Schmidt et al. (2024) [1], on the potential protective effect of cholesteryl ester transfer protein (CETP) inhibition on the risk of dementia. Using drug target Mendelian randomization, the authors examined the causal effects of CETP inhibition on total brain volume, white matter hyperintensity volume, Lewy body and Parkinson’s dementia as well as other cardiovascular traits. They concluded that, based on their findings, CETP inhibition may be a viable strategy to treat dementia.

Alzheimer’s disease (AD) is the most prevalent cause of dementia globally, responsible for around 80% of all dementia cases (compared to around 5% for Lewy body dementia) [2, 3]. Thus, it is surprising that, despite having several very large, publicly available genome-wide association studies (GWAS) for Alzheimer’s disease [4], this outcome was not considered. In a previous publication by the same authors in September 2021 [5], they showed little evidence of an effect of CETP inhibition on risk of AD in their main analysis, in which genetic variants instrumented CETP concentration (OR: 0.99, 95% confidence intervals [CI]: 0.91–1.07). In their additional analyses whereby the effect of CETP inhibition was scaled to the canonical drug effect of increasing high-density lipoprotein cholesterol (HDL), and decreasing low-density lipoprotein cholesterol (LDL) and triglycerides, there was even some tentative evidence of a harmful effect of CEPT inhibition on AD risk (scaled to increase in HDL OR: 1.04, 95% CI: 0.97–1.10; scaled to decrease in LDL OR: 1.63, 95% CI: 1.06–2.49; scaled to decrease in triglycerides OR: 1.36, 95% CI: 0.98–1.90).

**Fig. 1:**
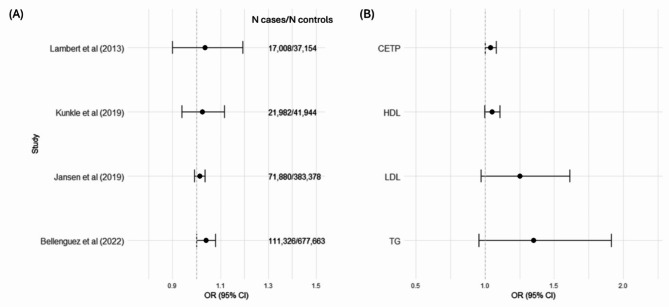
Panel (**A**) compares results of the causal effect of CETP inhibition on AD risk when using four different GWAS for AD. Panel (**B**) compares results of the causal effect of CETP inhibition on AD risk when scaling effects to CETP concentrations, increasing HDL cholesterol, and decreasing LDL cholesterol and triglycerides (TG), using the largest AD GWAS as the outcome (Bellenguez et al., 2022)

It is worth noting that in Schmidt et al. (2021) paper, the authors used the oldest GWAS of AD from 2013 [6]. Several larger GWAS meta-analyses have since been published [4, 7, 8]. For comparison, the AD GWAS used in the Schmidt et al. (2021) paper had 17,008 clinical cases and 37,154 controls [6]. The most recent AD GWAS has 39,106 clinically diagnosed cases, 46,828 ‘proxy’ cases and 401,577 controls [4]. Results with three larger GWAS are presented in Fig. 1 (panel A, all methods in supplement). Results are generally in agreement across the different GWAS but, as expected, there is greater precision when using in the largest. These findings suggest tentative evidence of a modest harmful effect of CETP inhibition on AD risk. Results scaled to the downstream biomarkers (HDL, LDL, and triglycerides) with the largest AD GWAS (Bellenguez et al., 2022 [4]) are also presented in Fig. 1 (panel B). Generally all analyses suggest either a null or a modest harmful effect on AD risk.

Cerebrovascular disease is the second most prevalent cause of dementia, present in over 70% of all dementia cases^2^. The authors identified evidence to suggest CETP inhibition may reduce risk of any stroke, ischemic stroke, and small vessel stroke, but there was little evidence of an effect on white matter hyperintensity volume; a clinical biomarker of small vessel disease. When examining the effect of CETP inhibition on risk of vascular dementia and all-cause dementia using the largest GWAS to date [9], there is very little evidence of an effect (vascular dementia OR: 1.11, 95% CI: 0.97–1.27) (all-cause dementia OR: 1.01, 95% CI: 0.95–1.07).

In conclusion, based on human genetic evidence, CETP is unlikely to be a viable target to treat, by far, the most prevalent causes of dementia.

## Electronic supplementary material

Below is the link to the electronic supplementary material.

Supplementary Material 1

## Data Availability

All data used in this study are publicly available. The data can be downloaded from the links shown in Table 1 of the online supplement (either from the online supplement of the original GWAS or in a linked online repository). The code used to run the analysis is provided at this link: https://github.com/emmylooroll/schmidt2024.

## Abbreviations

AD: Alzheimer’s disease
CETP: Cholesteryl ester transfer protein
CI: Confidence interval
GWAS: Genome-wide association study
HDL: High-density lipoprotein cholesterol
LDL: Low-density lipoprotein cholesterol
OR: Odds ratio
TG: Triglycerides
