# Supplementary material for: Response to Schmidt et al.: Lower activity of cholesteryl ester transfer protein (CETP) and the risk of dementia: a Mendelian randomization analysis
Source: Alzheimers Res Ther. 2024 Dec 19;16:264. doi: 10.1186/s13195-024-01631-4 (PMC11660916; doi:10.1186/s13195-024-01631-4)
Supplement: Supplementary file 1 — Supplementary Material 1 [file 13195_2024_1631_MOESM1_ESM.docx]

**Online methods for response to Schmidt et al.**

**Table 1: Data used in this paper**

| **Trait** |  | **URL** |
| --- | --- | --- |
| CETP | Blauw et al (2018) | **https://pubmed.ncbi.nlm.nih.gov/29728394/** |
| HDL | Willer et al (2013) | **https://pubmed.ncbi.nlm.nih.gov/24097068/** |
| LDL |  |  |
| Triglycerides |  |  |
| Alzheimer’s disease | Lambert et al (2013) | **https://pubmed.ncbi.nlm.nih.gov/24162737/** |
| Alzheimer’s disease | Kunkle et al (2019) | **https://pubmed.ncbi.nlm.nih.gov/30820047/** |
| Alzheimer’s disease | Jansen et al (2019) | **https://pubmed.ncbi.nlm.nih.gov/30617256/** |
| Alzheimer’s disease | Bellenguez et al (2022) | **https://pubmed.ncbi.nlm.nih.gov/35379992/** |
| All cause dementia | Fongang et al (2024) | **https://pubmed.ncbi.nlm.nih.gov/39046104/** |
| Vascular dementia |  |  |

***Main analysis scaled to CETP concentrations***the TwoSampleMR package was used to perform all analyses^1^. Genetic variants for CETP were identified from Blauw et al. (ref). Three SNPs reached genome-wide significance and were independent after clumping using an r^2^ threshold of 0.01, a 10,000 kb window, and a European reference panel (1000 Genomes) (Table 2). Associations between these genetic variants and circulating CETP concentrations in the blood were extracted from the GWAS summary statistics. The same three genetic variants were then looked up in several Alzheimer’s disease GWAS summary statistics (Table 1) to obtain the effect of the CETP variants on AD risk. Harmonisation was performed so that effect alleles were matched between the CETP and AD GWAS summary statistics (or flipped if they did not match). The SNP-exposure and SNP-outcome coefficients were combined using an inverse-variance-weighted (IVW) approach to give an overall estimate of the causal effect across all three CETP SNPs. The estimator is a Wald ratio and is equivalent to a weighted regression of the SNP-outcome coefficients on the SNP-exposure coefficients with the intercept constrained to zero. It is important to note that, to reflect the effect of CETP inhibitors (i.e. CETP lowering drugs), causal effect estimates from the MR analyses need to be flipped because by default in the TwoSampleMR package, they reflect an increase in the exposure (i.e. higher levels of circulating CETP), irrespective of the direction in which they are encoded in the input exposure file. The same harmonization and analysis process was conducted with all-cause dementia and vascular dementia as outcomes, using the data in Table 1.

***Scaling CETP to its effects on HDL, LDL and triglycerides***The three CETP variants were looked up in the HDL, LDL and triglycerides GWAS by Willet et al (Tables 3-5) to estimate the effect of CETP on HDL, LDL and triglycerides. This was then used as the exposure in the MR, instead of associations of the CETP variants on CETP concentrations, so that causal effects are scaled to an increase in HDL, and a decrease in both LDL and triglycerides. Again, to reflect the effect of CETP inhibitors (i.e. CETP lowering drugs) on raising HDL, and lowering LDL and triglycerides, causal effect estimates from the MR analyses of LDL and triglycerides need to be flipped to reflect an increase in those traits.

**Table 2: CETP variants used in all analyses and their effect on CETP concentrations**

| **CHR** | **SNP** | **POS** | **Effect allele** | **Other allele** | **EAF** | **BETA** | **SE** | **P** |
| --- | --- | --- | --- | --- | --- | --- | --- | --- |
| 16 | rs247616 | 56989590 | C | T | 0.67 | 0.32 | 0.015 | 3.98E-100 |
| 16 | rs12720922 | 57000885 | A | G | 0.17 | 0.35 | 0.019 | 3.48E-74 |
| 16 | rs1968905 | 57010948 | G | T | 0.82 | 0.12 | 0.02 | 4.12E-09 |

**Table 3: CETP variants used in all analyses and their effect on HDL concentrations**

| **CHR** | **SNP** | **POS** | **Effect allele** | **Other allele** | **EAF** | **BETA** | **SE** | **P** |
| --- | --- | --- | --- | --- | --- | --- | --- | --- |
| 16 | rs247616 | 56989590 | T | C | 0.29 | 0.24 | 0.004 | 1.00000e-200 |
| 16 | rs12720922 | 57000885 | A | G | 0.21 | -0.26 | 0.006 | 1.00000e-200 |
| 16 | rs1968905 | 57010948 | T | G | 0.19 | 0.06 | 0.008 | 1.09094e-15 |

**Table 4: CETP variants used in all analyses and their effect on LDL concentrations**

| **CHR** | **SNP** | **POS** | **Effect allele** | **Other allele** | **EAF** | **BETA** | **SE** | **P** |
| --- | --- | --- | --- | --- | --- | --- | --- | --- |
| 16 | rs247616 | 56989590 | T | C | 0.29 | -0.05 | 0.004 | 2.56626e-37 |
| 16 | rs12720922 | 57000885 | A | G | 0.21 | 0.05 | 0.007 | 1.42298e-13 |
| 16 | rs1968905 | 57010948 | T | G | 0.19 | -0.01 | 0.008 | 1.50000e-01 |

**Table 5: CETP variants used in all analyses and their effect on triglyceride concentrations**

| **CHR** | **SNP** | **POS** | **Effect allele** | **Other allele** | **EAF** | **BETA** | **SE** | **P** |
| --- | --- | --- | --- | --- | --- | --- | --- | --- |
| 16 | rs247616 | 56989590 | T | C | 0.29 | -0.04 | 0.004 | 1.12305e-25 |
| 16 | rs12720922 | 57000885 | A | G | 0.21 | 0.04 | 0.006 | 3.23519e-11 |
| 16 | rs1968905 | 57010948 | T | G | 0.19 | -0.007 | 0.007 | 4.79400e-01 |

1. Gibran Hemani JZ, Kaitlin H Wade, Charles Laurin, Benjamin Elsworth, Stephen Burgess, Jack Bowden, Ryan Langdon, Vanessa Tan, James Yarmolinsky, Hashem A. Shihab, Nicholas Timpson, David M Evans, Caroline Relton, Richard M Martin, George Davey Smith, Tom R Gaunt, Philip C Haycock. MR-Base: a platform for systematic causal inference across the phenome using billions of genetic associations. *bioRxiv* 2017.
